# Controllable Preparation of Cubic Zeolite A and Application of Langmuir Model in Carbon Dioxide Adsorption

**DOI:** 10.3390/nano11123375

**Published:** 2021-12-13

**Authors:** Peng Wang, Jun Cao, Yujiao Zhang, Qi Sun

**Affiliations:** 1 College of Materials and Metallurgy, Guizhou University, Huaxi District, Guiyang 550025, China; pengwang180907@163.com (P.W.); juncaowyy@163.com (J.C.); 2 Center for R & D of Fine Chemicals, Guizhou University, Huaxi District, Guiyang 550025, China; zhangyujiao105@163.com

**Keywords:** fly ash, zeolite A, Langmuir model, carbon dioxide

## Abstract

A large amount of remaining fly ash has been piled up or landfilled, which not only a waste of land resources but also results in a series of environmental problems. Therefore, using fly ash to produce high value-added products is a win-win development orientation between human beings and nature. In this study, zeolite A is successfully synthesized using a hydrothermal method using fly ash. Additionally, it is at 1.0 mol·L^−1^ of the alkali concentration that the crystallinity of zeolite A reaches the maximum value, about 96.6%. FTIR research shows that the main secondary structural unit D4R vibration band of zeolite A appears at 555 cm^−1^. The results of the SEM study indicate the structure of zeolite A is cubic. The TEM results show that the crystal structure of the zeolite A belongs to the body-centered cubic structure. Meanwhile, the positively charged sodium ions cooperate with the silicon oxygen tetrahedron and the aluminum oxygen tetrahedron to form the zeolite A skeleton. Carbon dioxide adsorption equilibrium study shows that the maximum adsorption capacity of zeolite A of 46.5 mL·g^−1^ is significantly higher than the maximum adsorption capacity of commercial-grade zeolite 4A of 39.3 mL·g^−1^. In addition, the application of the Langmuir model in the adsorption of carbon dioxide by commercial-grade zeolite 4A and zeolite A is studied, which not only extends the application of zeolite A, but can be further extended to other zeolite materials as well. Meanwhile, the adsorption process belongs to the Langmuir model, which is a single layer adsorption on an ideal surface.

## 1. Introduction

As a typical kind of fossil fuel, coal is distributed widely in the world [1,2,3]. With the rapid development of the global economy, the demand for energy is growing rapidly. Therefore, the amount of coal consumed for power generation and industrial combustion is increasing rapidly, and a large amount of fly ash is produced [4]. According to statistics, fly ash produced from coal-fired power generation is approximately 500 million tons each year in China, but about 50% of fly ash waste is underutilized and is piled up or buried in the garbage pool [5,6]. Fly ash contains trace amounts of toxic substances, such as arsenic, platinum, selenium, barium, chromium, copper, lead, platinum, nickel, vanadium and other heavy metals. Landfilling a large amount of fly ash will aggrandize these toxic substances [7], and once the leachate of the landfill leaks, it is doomed to cause a huge environmental risks and irreparable environmental disasters. Fly ash is a kind of solid waste which has become one of the serious pollution sources. It not only brings about atmospheric pollution, but threatens to human health and soil as well [8]. Therefore, it is urgent to think about recycling and reusing the fly ash.

Zeolite A, an inorganic material with a microporous structure, is a kind of aluminosilicate compound which equipped with many excellent characteristics such as non-toxic, high porosity, large specific surface area, good thermal stability, high cation exchange capacity and environmental friendliness [9,10,11,12,13]. It has a wide range of potential applications in many fields such as gas separation, hard water softening, wastewater treatment, catalytic cracking in the petrochemical industry, new functional materials and so on [14,15,16]. Recently, researchers discovered that the Al_2_O_3_ and SiO_2_ is the main components of fly ash which can provide aluminum and silicon sources for synthetic zeolite. Since Holler and Wirsching [17] successfully obtained zeolite from fly ash for the first time, great effort has been taken in the preparation of zeolites by the hydrothermal method. Liu et al. [18] reported that using fluidized bed fly ash as the raw material, a solvent-free method was used to synthesize zeolite P and the research on the removal of ammonia in water is carried out. Wu et al. [19] proposed that coal gasification fine slag is used as a low-cost raw material to prepare the P-zeolite/carbon composite in situ, and the adsorption and removal of crystal violet on the composite material is studied. In addition, Ariful et al. [20] reported that the adsorption equilibrium of zeolite A on carbon dioxide. The obtained isotherm data is correlated as a function of temperature and pressure to fit with Langmuir model equation.

In this study, zeolite A is successfully synthesized by hydrothermal method using fly ash as a raw material. The zeolite A is characterized in detail by powder XRD, FTIR, SEM, TEM, and carbon dioxide analyzer. The effect of alkali concentration on crystal nucleation and crystal growth during the synthesis of zeolite was studied. The application of Langmuir model in the adsorption of carbon dioxide by zeolite A and commercial-grade zeolite 4A was systematically studied.

## 2. Materials and Methods

### 2.1. Materials

**Figure A1:**
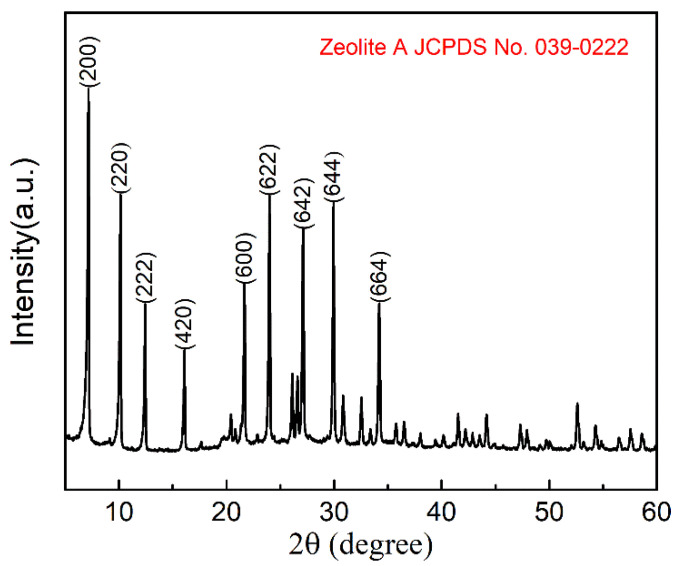
XRD pattern of commercial-grade zeolite 4A.

**Figure A2:**
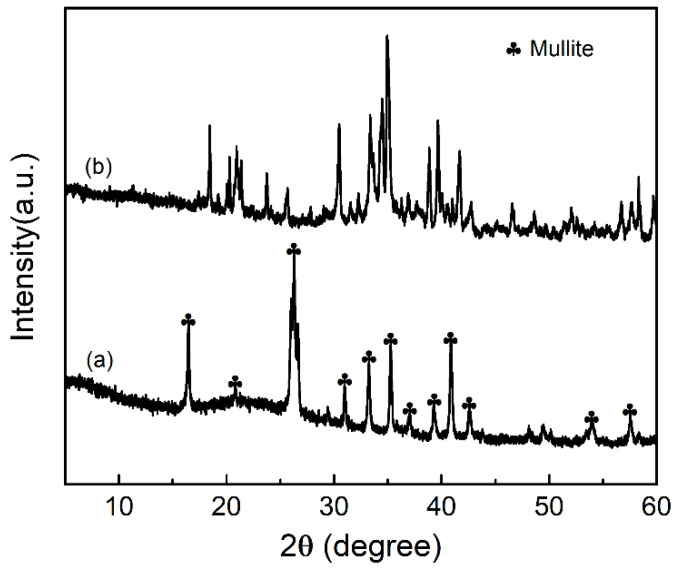
XRD patterns of fly ash (**a**) and activated fly ash (**b**).

**Figure A3:**
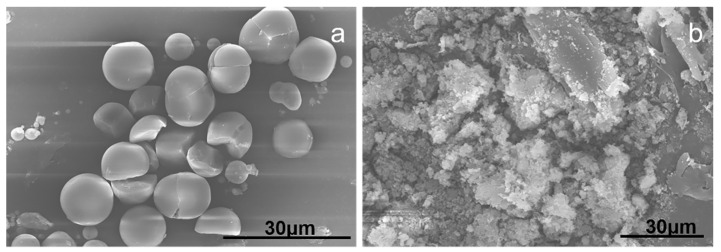
SEM images of fly ash (**a**) and activated fly ash (**b**).

The fly ash was provided by a thermal power plant (Inner Mongolia Thermal Power Plant, Inner Mongolia, China) in China. Fly ash which used in the experiment is mainly composed of 46.36 wt.% SiO_2_ and 45.08 wt.% Al_2_O_3_. Moreover, the Si/Al molar ratio is around 1.0, there are no extra Si- or Al-sources needed. The main crystalline component of fly ash is mullite, as shown in Figure A2a. The silicon and aluminum sources in fly ash are in an inactive state. The crucial step for activation treatment of fly ash is calcining mixtures which are consists of fly ash and sodium hydroxide at 600 °C for 2.5 h. XRD pattern of activated fly ash is shown in Figure A2b. The SEM images of fly ash and activated fly ash are shown in Figure A3. The chemical reagents (NaOH) used were analytically pure and purchased from Sinopharm Chemical Reagent Co., Ltd. (Shanghai, China). Commercial-grade zeolite 4A was purchased from Sinopharm Chemical Reagent Co., Ltd. XRD pattern of the commercial-grade zeolite 4A is shown in Figure A1. Deionized water was prepared in the laboratory.

### 2.2. Synthesis of Zeolite A

**Figure 1:**
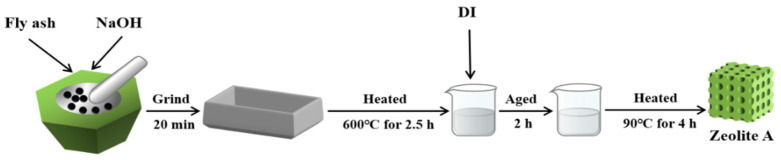
Synthetic diagram of zeolite A.

First of all, the pretreated fly ash and NaOH are mixed evenly according to a certain mass ratio. Then, the mixture was ground with an agate mortar for 20 min and heated at 600 °C for 2.5 h. After cooling to room temperature, the melt was dissolved in distilled water, fully stirred, aged for 2 h, and subjected to hydrothermal treatment. To explore the connections about NaOH concentration on the crystallinity and the purity of the prepared zeolite A, experiment was performed at 90 °C for 4 h transform the concentrations at a range of 0.25–2.5 mol·L^−1^ of NaOH. Finally, the synthesized zeolite A was filtered, washed, and dried at 60 °C for 10 h, and then further experiments and characterizations were performed. Figure 1 is a schematic diagram of the synthesis of zeolite A.

### 2.3. Characterization

The structural characteristics and crystallinity of the samples were analyzed with the German Bruker AXS D8-Focus X-ray diffractometer. The characteristic peaks of zeolite A appeared at 2θ = 7.10°, 10.08°, 12.40°, 16.08°, 21.60°, 24.12°, 27.04°, 29.86° and 34.10°. The sum ratio of the characteristic peak heights of the samples to the corresponding sum of the peak heights in the commercial-grade zeolite 4A was used to calculate the crystallinity. The crystallinity was calculated by Equation (1):

(1) $$\%\mathrm{Crystallinity}=\left(\frac{\sum^{\;}\mathrm{peak}\;\mathrm{height}\;\mathrm{of}\;\mathrm{sample}}{\sum^{\;}\mathrm{peak}\;\mathrm{height}\;\mathrm{of}\;\mathrm{commercial}-\mathrm{grade}\;\mathrm{zeolite}\;A}\right)\times 100\%$$

Perkin-Elmer infrared spectrometer was used to measure the Fourier transform infrared spectrum of the samples. The chemical composition of the sample is measured by wavelength dispersive X-ray fluorescence spectrometry on an Axios X-ray fluorescence spectrometer. Hitachi SU8010 scanning electron microscope is used to analyze the microscopic morphology of the samples. The Tecnai G2 F20 S-TWIN field emission transmission electron microscope used for the microstructure analysis of the samples is a multi-functional, multi-user environment 200 kV field emission transmission electron microscope. The nitrogen adsorption experiment was carried out on JW-BK200CJW-BK200C (Beijing Jingwei Gaobo Technology Co., Ltd., Beijing, China)). Before the test, the sample was degassed at 200 °C for 10 h, and the nitrogen adsorption-desorption measurement was carried out at liquid nitrogen temperature (77K). The Brunauer–Emmett–Teller (BET) method was used to calculate the surface area of the sample. The pore size distribution was studied by BJH method. The carbon dioxide adsorption equilibrium of the samples is studied on the scientific compass test platform. The carbon dioxide adsorption experiment is performed using an ASAP2460 instrument (Micromeritics Instrument Corp, Gwinnett County, GA, USA).

## 3. Results and Discussion

### 3.1. XRD Analysis

**Figure 2:**
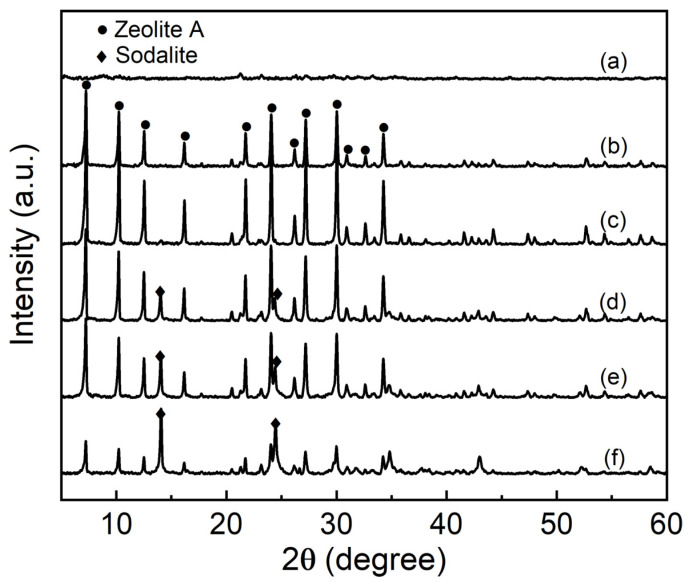
XRD patterns of samples synthesized at different NaOH concentrations (NaOH concentrations: (**a**) 0.25 mol·L^−1^, (**b**) 0.5 mol·L^−1^, (**c**) 1.0 mol·L^−1^, (**d**) 1.5 mol·L^−1^, (**e**) 2.0 mol·L^−1^ and (**f**) 2.5 mol·L^−1^).

Through the analysis of the experimental results, it can be clearly seen that the alkali concentration of the synthesis system plays an important role in the synthesis of zeolite A. Figure 2 presents the XRD patterns of samples synthesized at different NaOH concentrations. It can be seen from Figure 2 that when the alkali concentration is 0.25 mol·L^−1^, the XRD pattern shows amorphous characteristics which indicate that the element Si and Al in the sample did not form a specific chemical bond, and the binding ability was weak. In addition, the characteristic diffraction peaks of zeolite A do not appear at this alkali concentration. At this experiment condition, due to the alkali concentration is too low, the dissolution rate of the amorphous aluminosilicate precursor is slow, thus the concentration of active aluminate, silicate and aluminosilicate materials is low, which limits the formation of zeolite A crystal nuclei and crystal growth. With the increase of alkali concentration, when alkali concentration reaches 0.5 mol·L^−1^, the characteristic peaks for zeolite A are appeared at 2θ = 7.10°, 10.08°, 12.40°, 16.08°, 21.60°, 24.12°, 27.04°, 29.86° and 34.10° which are conformed to the reference values for single phase zeolite A according to the JCPDS NO. 039-0222 [21]. Subsequently, with the increase of alkali concentration, the intensity of the characteristic diffraction peaks first increases and then decreases. When the alkali concentration is 1.0 mol·L^−1^, the characteristic diffraction peak intensity of zeolite A reaches the largest as shown in Figure 2. However, when the alkali concentration is 1.5 mol·L^−1^, the zeolite A are partially transformed into hydroxyl sodalite. The thermodynamic stability of zeolite A is less than that of hydroxyl sodalite, as a result, the zeolite A formed in the first step spontaneously transformed into thermodynamically more stable hydroxyl sodalite.

**Figure 3:**
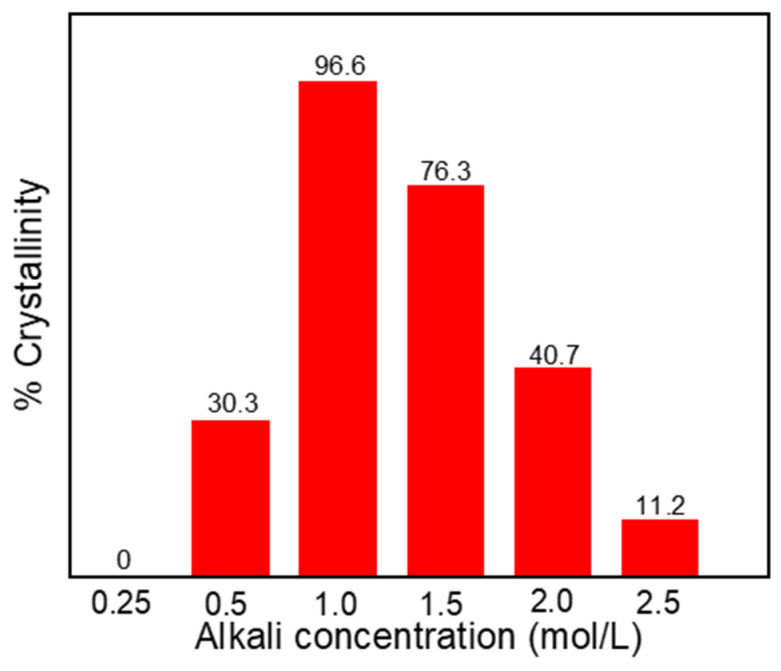
The crystallinity of samples synthesized at different NaOH concentrations of 0.25 mol·L^−1^, 0.5 mol·L^−1^, 1.0 mol·L^−1^, 1.5 mol·L^−1^, 2.0 mol·L^−1^ and 2.5 mol·L^−1^.

Figure 3 is the crystallinity of samples synthesized at different NaOH concentrations of 0.25 mol·L^−1^, 0.5 mol·L^−1^, 1.0 mol·L^−1^, 1.5 mol·L^−1^, 2.0 mol·L^−1^ and 2.5 mol·L^−1^. It can be seen from Figure 3 that when the alkali concentration of the solution is in 0.25–1.0 mol·L^−1^, the crystallinity of the samples gradually increases with the increase of alkali concentration, furthermore when the alkali concentration is 1.0 mol·L^−1^, the crystallinity of zeolite A reaches the maximum value, about 96.6%. Nevertheless, as the alkali concentration continues to increase, the crystallinity of the samples gradually decreases. When the alkali concentration increased to 2.5 mol·L^−1^, the crystallinity of zeolite A decreased to 11.2%. The research results show that proper alkali concentration accelerates crystal growth and makes the crystal crystallize completely. Due to zeolites being in a metastable state, too high a concentration of alkali will dissolve zeolite A in the hot alkali solution condition, resulting in a decrease in crystallinity. Therefore, an appropriate alkali concentration is beneficial to maintain the stability of zeolite A crystals.

### 3.2. FTIR Analysis

**Figure 4:**
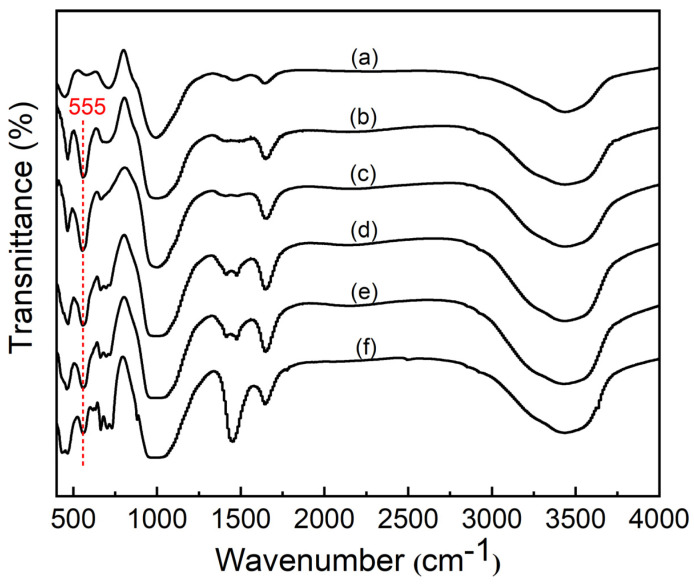
FTIR spectra of samples synthesized at different NaOH concentrations (NaOH concentrations: (**a**) 0.25 mol·L^−1^, (**b**) 0.5 mol·L^−1^, (**c**) 1.0 mol·L^−1^, (**d**) 1.5 mol·L^−1^, (**e**) 2.0 mol·L^−1^ and (**f**) 2.5 mol·L^−1^).

The FTIR spectra of samples synthesized at different NaOH concentrations are shown in Figure 4. All the samples exhibit absorption bands around 3435 cm^−1^ and 1654 cm^−1^ assigning to O-H vibrations of the absorbed water. The bands around 994 cm^−1^, 730 cm^−1^ and 450 cm^−1^, which belong to sodium aluminosilicate gels, appeared when the alkali concentration is 0.25 mol·L^−1^. These bands are due to the asymmetric stretching vibration of internal tetrahedra, the symmetric stretching vibration and the bending vibration modes of T-O bonds in TO4 tetrahedra (where T = Si or Al), respectively. When the alkali concentration reaches 0.5 mol·L^−1^, the absorption band formed by the vibration of the Si-O-Si group around 1005 cm^−1^ shifts slightly, which indicated that the octahedral Al transformed into tetrahedral Al in the sodium aluminosilicate gels, and part of tetrahedral Al combined with Si-O-Si bond forming Si-O-Al bond. The band at 555 cm^−1^ assigned to D4R vibration which is the major secondary building unit of zeolite A, appeared when the alkali concentration reached to 0.5 mol·L^−1^ [22,23]. Furthermore, the 555 cm^−1^ band is increased with increasing alkali concentration. When the alkali concentration is 1.0 mol·L^−1^, this band has the highest intensity.

### 3.3. SEM Analysis

**Figure 5:**
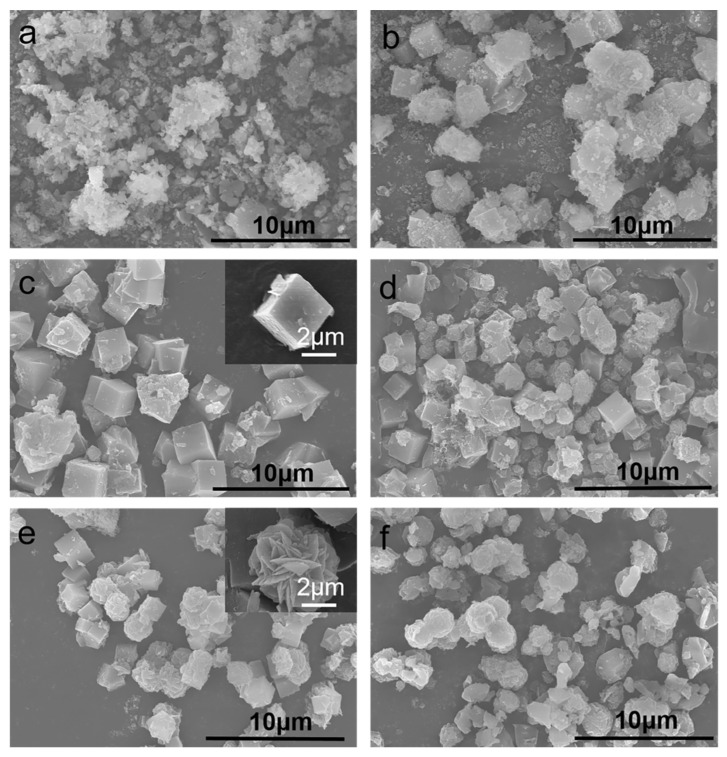
SEM images of samples synthesized at different NaOH concentrations (NaOH concentrations: (**a**) 0.25 mol·L^−1^, (**b**) 0.5 mol·L^−1^, (**c**) 1.0 mol·L^−1^, (**d**) 1.5 mol·L^−1^, (**e**) 2.0 mol·L^−1^ and (**f**) 2.5 mol·L^−1^).

The SEM images of samples synthesized at different NaOH concentrations are shown in Figure 5. When the alkali concentration is 0.25 mol·L^−1^, the sample shows the appearance of amorphous aluminosilicate, indicating that zeolite A has not started to crystallize. Under this alkali concentration, the hydroxide ion concentration is too low, the dissolution rate of amorphous aluminosilicate is slow, and the concentration of active aluminate, silicate and aluminosilicate is low, which is not conducive to the formation of crystal nuclei. In addition, the low concentration of cations (Na^+^) inhibits the formation of the zeolite A framework structure. As the alkali concentration increases, zeolite A begins to crystallize. When the alkali concentration is 0.5 mol·L^−1^, the cubic zeolite A appears on the surface of the amorphous aluminosilicate structure. When the alkali concentration is 1.0 mol·L^−1^, the amorphous aluminosilicate structure disappears completely, and the cubic zeolite A has a good microscopic crystal morphology (Figure 5c (inset)). Therefore, it can be concluded that as the alkali concentration increases, the nucleation rate in the synthesis system increases and the polymerization rate between silicate ions and aluminate ions increases. However, as the alkali concentration increases, sodalite will be formed in the synthetic system. An enlarged view of sodalite is shown in Figure 5e (inset). Next, a systematic study of zeolite A synthesized under optimal conditions is carried out.

### 3.4. TEM Analysis

**Figure 6:**
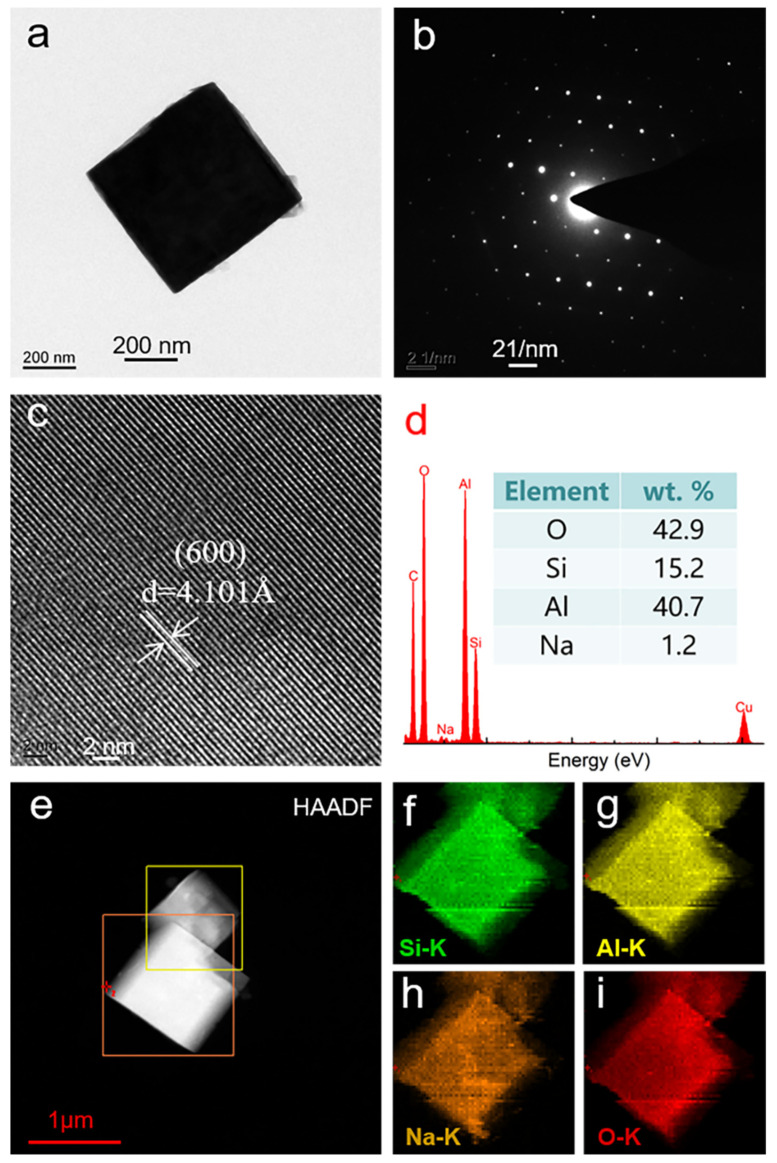
TEM analysis results of zeolite A. (**a**) TEM micrograph, (**b**) SAED, (**c**) lattice fringes, and (**d**) EDX pattern and element content (inset); HAADF STEM- EDX elemental spectrum of zeolite A. (**e**) selected areas, (**f**) Si element distribution, (**g**) Al element distribution, (**h**) Na element distribution, (**i**) O element distribution.

The microstructure of zeolite A was further studied by the TEM technique, as shown in Figure 6. Figure 6a shows that the morphology of zeolite A is consistent with the standard zeolite A in size and structure, indicating that zeolite A has the characteristics of solid growth and opaque morphology. Figure 6b is an electron diffraction pattern, indicating that zeolite A has a body-centered cubic structure. The HRTEM image (Figure 6c) shows clear lattice fringes with a crystal interplanar spacing of about 0.4101 nm, which corresponds to the (600) crystal plane of zeolite A. The EDX analysis results (Figure 6d) show that the main constituent elements are Na, Al, Si and O, indicating that the elements Si, Al, Na and O constitute the framework of zeolite A. According to the element content table, the percentages of Na, Al, Si and O in zeolite A are 1.2%, 40.7%, 15.2% and 42.9%, respectively. The HAADF STEM-EDX element mapping is used to further reveal the distribution of Si, Al, Na and O elements in the zeolite A structure. From Figure 6e–i, it can be more intuitively observed that Si, Al, Na and O elements are distributed in the zeolite A crystal. In addition, it can be seen that the elements coexist and are uniformly distributed. Meanwhile, sodium ions cooperate with silicon-oxygen tetrahedra and aluminum-oxygen tetrahedra to form the framework structure of zeolite A.

### 3.5. BET Analysis

**Figure 7:**
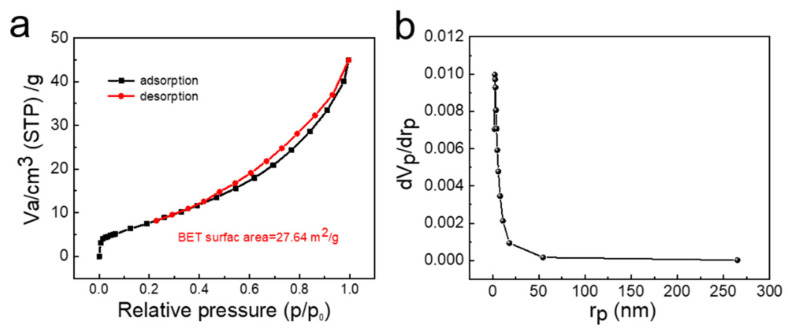
(**a**) N_2_ adsorption-desorption isotherms and (**b**) pore size distribution of zeolite A.

The specific surface area and pore size distribution of zeolite A were studied using N_2_ adsorption-desorption technology. In the range of relative pressure P/P_o_ = 0.5–1.0, pressure hysteresis is observed (Figure 7a), indicating that the sample contains a mesoporous structure [24,25,26,27,28]. The Brunauer–Emmett–Teller (BET) surface area of zeolite A obtained by BET method is 27.64 m^2^·g^−1^. The Barrett–Joyner–Halenda equation (BJH) method is used to study the pore size distribution of the zeolite A as shown in Figure 7b.

### 3.6. Carbon Dioxide Adsorption Analysis

The Langmuir model is usually used to study the adsorption-desorption behavior of zeolite A at different carbon dioxide concentrations at specific temperatures. This model shows that the adsorption rate of carbon dioxide on the zeolite A surface is proportional to the carbon dioxide concentration. In addition, the carbon dioxide concentration is directly proportional to the pressure under certain conditions. By considering that the total surface coverage is equal to unity, the occupied fractional surface coverage is *θ* and the unoccupied fractional surface coverage is 1 − *θ* [29]. Therefore, the dynamic interaction between carbon dioxide and zeolite A can be expressed by Equation (2) [30,31]:

(2) $$\frac{d\theta \left(t\right)}{dt}=k_{ads}C\left[1-\theta \left(t\right)\right]-k_{des}\theta \left(t\right)$$

The first and second terms on right hand side of Equation (2) are the adsorption and desorption rates, and *k_ads_* and *k_des_* are the adsorption and desorption coefficients, respectively. Assuming the surface coverage is linearly proportional to the carbon dioxide adsorption capacity (*Q*), Equation (2) can be rewritten as:

(3) $$\frac{dQ\left(t\right)}{dt}=k_{ads}C\left[\beta -Q\left(t\right)\right]-k_{des}Q\left(t\right)$$

Here, *β* is the constant of proportionality between the surface coverage and the amount of carbon dioxide adsorption. To solve Equation (3), suppose that all surface adsorption sites are not occupied by carbon dioxide at *t* = 0 (*Q* = 0), and all surface adsorption sites are completely occupied by carbon dioxide at *t*→∞ (*Q* = *Q_st_*), the carbon dioxide adsorption capacity is expressed as:

(4) $$Q\left(t\right)=Q_{st}\left(1-e^{-\left(k_{ads}C+k_{des}\right)t}\right)$$

The equilibrium adsorption capacity (*Q_st_*) is given by the following formula:

(5) $$Q_{st}=\frac{\beta k_{ads}C}{k_{ads}C+k_{des}}$$

**Figure A4:**
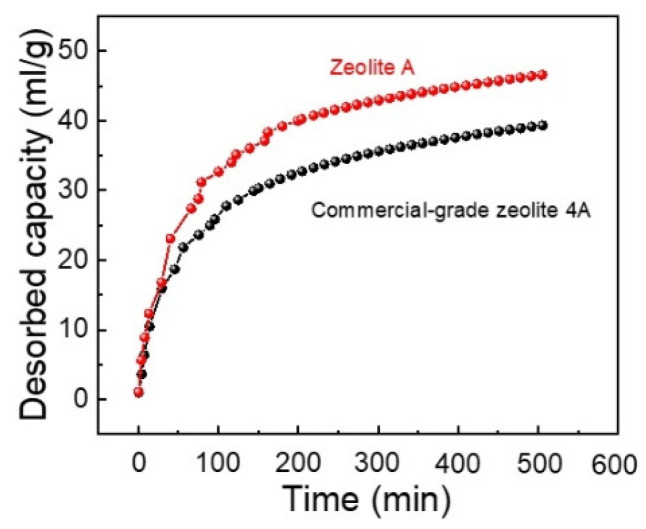
The relationship between carbon dioxide adsorption capacity and time.

When the equilibrium state is reached, that is, when the adsorption and desorption rates are equal, equilibrium adsorption will occur. Meanwhile, the Langmuir model can describe the initial adsorption state during the adsorption process. From Figure A4, it can be seen that the initial adsorption state is consistent with Equation (4). Therefore, at a small interval (*t*→0), *e^−(kads C + kdes) t^*≈ *1* *− (k_ads_ C + k_des_) t*, Equation (4) can be approximated as:

(6) $$Q\left(t\right)=\beta k_{ads}Ct$$

The carbon dioxide adsorption capacity in Equation (6) changes with time in a positive gradient. Equations (5) and (6) can be simply expressed as:

(7) $$Q_{st}=\frac{\beta }{1+\frac{1}{KC}}$$

(8) $$\frac{dQ\left(t\right)}{dt}=\beta k_{ads}C$$

Among them, *K* is the equilibrium constant, which is determined by *k_ads_/k_des_*.

**Figure 8:**
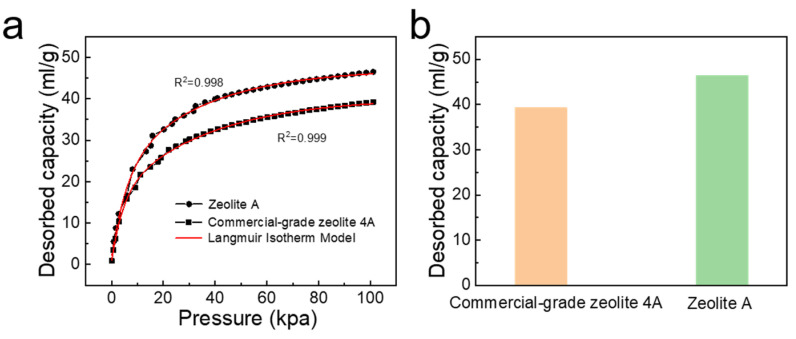
(**a**) Carbon dioxide adsorption isotherm of commercial-grade zeolite 4A and zeolite A at 298 K: Langmuir model fitting; (**b**) the histogram of maximum adsorption amount for carbon dioxide: commercial-grade zeolite 4A and zeolite A.

Figure 8a is carbon dioxide adsorption isotherm of commercial-grade zeolite 4A and zeolite A at 298 K. From Figure 8a, as the equilibrium pressure increases, the amount of carbon dioxide adsorbed rapidly increases, indicating that an appropriate equilibrium pressure promotes the activity of most adsorption sites. As the equilibrium pressure continues to increase, the amount of carbon dioxide adsorption tends to stabilize, indicating that all adsorption sites are occupied by carbon dioxide. In addition, the initial slope has a linear relationship with the carbon dioxide concentration, and then it exhibits saturation behavior at high carbon dioxide concentrations. Meanwhile, the determination coefficient values of the Langmuir model of zeolite A and commercial-grade zeolite 4A are 0.998 and 0.999, respectively. Therefore, the experimental data in Figure 8a is in good agreement with the Langmuir model represented by Equation (7). In general, combined with the experimental data, the carbon dioxide adsorption characteristics of zeolite A are in good agreement with the Langmuir adsorption model, which proves the effectiveness of the model to describe the carbon dioxide adsorption characteristics of zeolite A. It is concluded from the above research that this study has guiding significance for determining the adsorption pressure and regeneration pressure in the pressure swing adsorption process. Meanwhile, the adsorption process is a single layer adsorption on an ideal surface [32]. In addition, when carbon dioxide adsorption reaches an equilibrium state, the maximum adsorption capacity of zeolite A is 46.5 mL·g^−1^, compared to 39.3 mL·g^−^^1^ of the commercial-grade zeolite 4A (Figure 8b). Consequently, zeolite A synthesized with fly ash has certain advantages in carbon dioxide adsorption applications.

## 4. Conclusions

In this work, zeolite A with fly ash as raw material is synthesized and the influence of the alkali concentration of the synthesis system on the crystallization of zeolite A is studied. It is found that during the synthesis of zeolite A, alkali concentration can improve the crystallization rate by promoting the formation of crystal nucleus and crystal growth. On the other hand, the introduction of OH^-^ ions into the reaction system must also introduce corresponding cations (Na^+^). Cations play an important role in the polycondensation reaction of aluminosilicate. Meanwhile, cations have an important influence on the polymerized state of silicate and the colloidal chemistry of aluminosilicate. The cations also play a very important role in the formation of zeolite A framework structure. In addition, the application of Langmuir model in the adsorption of carbon dioxide by commercial-grade zeolite 4A and zeolite A is studied. Combined with the experimental data, the carbon dioxide adsorption characteristics of commercial-grade zeolite 4A and zeolite A are in good agreement with the Langmuir adsorption model, which proves that the adsorption process is a single layer adsorption on an ideal surface.

## Data Availability

The study did not report any data.
